# Connectivity of the Superficial Muscles of the Human Perineum: A Diffusion Tensor Imaging-Based Global Tractography Study

**DOI:** 10.1038/s41598-018-36099-4

**Published:** 2018-12-14

**Authors:** Ali Zifan, Marco Reisert, Shantanu Sinha, Melissa Ledgerwood-Lee, Esther Cory, Robert Sah, Ravinder K. Mittal

**Affiliations:** 1 Department of Bioengineering, Medicine & Radiology, University of California, San Diego, CA USA; 2 Department of Radiology, Medical Physics, Faculty of Medicine, University Hospital Freiburg, Freiburg, Germany

## Abstract

Despite the importance of pelvic floor muscles, significant controversy still exists about the true structural details of these muscles. We provide an objective analysis of the architecture and orientation of the superficial muscles of the perineum using a novel approach. Magnetic Resonance Diffusion Tensor Images (MR-DTI) were acquired in 10 healthy asymptomatic nulliparous women, and 4 healthy males. Global tractography was then used to generate the architecture of the muscles. Micro-CT imaging of a male cadaver was performed for validation of the fiber tracking results. Results show that muscles fibers of the external anal sphincter, from the right and left side, cross midline in the region of the perineal body to continue as transverse perinea and bulbospongiosus muscles of the opposite side. The morphology of the external anal sphincter resembles that of the number ‘8’ or a “purse string”. The crossing of muscle fascicles in the perineal body was supported by micro-CT imaging in the male subject. The superficial muscles of the perineum, and external anal sphincter are frequently damaged during child birth related injuries to the pelvic floor; we propose the use of MR-DTI based global tractography as a non-invasive imaging technique to assess damage to these muscles.

## Introduction

The superficial pelvic floor muscles or perineal muscles are located inferior/caudal to the deep pelvic floor muscles, and comprise of external anal sphincter, transverse perinea, bulbospongiosus and ischiocavernosus muscles^1,2^. Considerable controversy continues to surround the nomenclature and function of these muscles^3^. Identified originally by surgical dissection in the cadavers, a number of imaging techniques such as computed tomography (CT), magnetic resonance imaging (MRI), and ultrasound imaging have been used to visualize these muscles in live human subjects. The emphasis of these imaging techniques is to visualize the boundaries of these muscles, and not the direction of individual muscle fibers in the muscle (myoarchitecture). High frequency ultrasound imaging has been used to visualize the muscle architecture of the superficial muscles of the leg, but there are no such studies in the perineum^4^.

Diffusion Tensor Imaging (DTI) and tractography provides information on the characteristic tissue composition, architectural organization, and physical properties of the tissue^5,6^. One of the advantages of diffusion tensor imaging (DTI) is its capability to determine fiber tracks in a noninvasive manner^7^. DTI based tractography has been used extensively to visualize the white matter tracts in the brain. However, DTI has much lower signal-to-noise ratio in muscles compared to the brain, because of a much shorter transverse relaxation (T2). Hence, there are only a limited number of studies to date of DTI based fiber tractography in the muscles^8–18^, specifically the pelvic floor muscles^19–26^. Multi-pennate structure of muscle groups of the lower leg^5^, the change of pennation angle with ankle rotation^27^ and changes in diffusion tensor indices with age and limb disuse^28^ using diffusion-based deterministic fiber tracking have been described in the literature.

Fiber reconstruction methods in DTI can be categorized into local and global approaches^29^. Several local approaches based on the principle of fiber tracking such as streamline propagation (SP)^30^, tensor deflection (TD)^31^, and the FACT algorithm^32^ have been devised. These approaches make restricted use of local tensor information, by deterministically computing fiber direction at each considered point, and then propagating fibers sequentially in independent steps. Such deterministic fiber tracking approaches, which has been used for muscle fiber tracking in the literature, is inherently an ill-posed inverse problem, as small errors introduced during the step-by-step reconstruction of a fiber bundle trajectory can lead to large errors in the evaluation of local fiber orientations^6^. The latter becomes even more complex, when there exist a myriad of fibers passing through a single voxel following different directions, as may be the case in the muscles of the perineum, especially the perineal body^19^. Thus, a general limitation of this concept is that minor inaccuracies in the chain of local decisions can accumulate, and affect the final result significantly.

The goal of our study was to construct the myoarchitecture of the superficial pelvic floor muscles, especially the perineal body, where fibers cross from one side of the body to the other, using a Bayesian framework incorporating stochastic modeling to carry out a global diffusion-based search via Gibbs sampling^7^. The latter approach has been applied in the brain to visualize crossing white matter tracts. The objective of the optimization is to find a segment configuration, which explains the signal as best as possible. The segments can bind to each other to form long chains, representing a streamline/fiber bundle. The prior energy controlling the connection behavior influences the reconstruction in such a way that meaningful fiber configurations are preferred. This idea helps to make the method robust and to cope with challenging low-quality data.

## Methods

### Subjects

We conducted a prospective study in which data were collected from ten healthy asymptomatic nulliparous females (28 ± 9 years), and four males (30 ± 6 years). Subjects filled out urinary and fecal continence questionnaires and were judged to have no symptoms related to pelvic floor dysfunction, especially urinary incontinence, anal incontinence or organ prolapse. The protocol for these studies was approved by the human Institutional Review Board (IRB) committee at the University of California San Diego (Protocol # 111030), and each subject signed an informed consent form prior to their enrollment in the study. All experiments were performed in accordance with relevant guidelines and regulations of the University of California, San Diego.

### Image Acquisition

The MRI scans were performed on a 3-T (GE Medical Systems, Milwaukee, WI) scanner systems, (Signa HDxt or MR 750) using an eight-channel, HD Cardiac phase-array coil (rather than Torso coil) to preserve the signal to noise ratio (SNR). The subjects were placed in the scanner with the feet-first-supine position. Extensive optimization of scanning parameters was carried out with respect to the signal to noise ratio (SNR), total acquisition time, spatial resolution, artifacts and image quality^19^. An initial 3-plane localizing scan was followed by a high resolution, axial morphological scan and finally the diffusion tensor (DT) scan. Morphology was found to be best delineated by the fat-saturated proton-density scan, with a 20 cm Field-of-view, 3 mm slice thickness, 0.4 mm slice separation and a matrix of 256 by 192. Our optimal protocol used a spin echo-based echo planar diffusion-weighted sequence with spatial spectral fat saturation, minimum possible echo time of 50–60 ms, repetition time of 4,500–6,500 ms, 20-cm field of view, 3-mm slice thickness, 0.4-mm spacing and 8 averages^19^. Different number of slices, 24 ~ 30 (depending on the length of the region of interest which varied among subjects) were acquired in the axial plane of the spine. In general, the SNR of DT imaging is significantly lower in the muscle than brain because of the lower spin-spin relaxation times (T2) in the muscle (about 35 msec) compared to that in the brain (about 90 msec). Based on our experience^19^, a b-value of 400 s/mm^2^ is optimal in various muscles. For every set, an intensity threshold was measured in the noise regions of the b = 0 image and used to remove background pixels from the computation. The SNR at various tissue points was measured as the ratio of the gray scale intensity in the b0 image of an ROI in the tissue of interest to the standard deviation of an ROI placed outside the image region of the anatomy. The b0 SNRs thus measured had a mean of 54.74 ± 26.6 in the subjects. Furthermore, across all the 32 directions, mean SNR was 31.02 ± 11.53. The number of diffusion-gradient directions was also optimized with respect to the quality of fiber tracking, 1 baseline and 32 non-collinear gradient directions were finally used.

## Data Analysis

### MRI Segmentation

All MRI Volumes were first imported into a computer software (AMIRA 6.0, Visage, Carlsbad, California). The perineum muscles (bulbospongiosus, superficial transverse perinea, external anal sphincter) were manually marked and outlined in the axial and coronal planes using b0 and colored FA maps, at their expected sites. The corresponding T2w anatomical series was used for anatomical reference. In some instances, based on initial fiber tracking results the masks were re-adjusted. This process was carried out for all the subjects in the dataset. The masks were subsequently imported into Matlab 2017b (The Mathworks, Inc.) to carry out fiber tracking and post processing.

### Global Tractography

We employed a global approach to study the connectivity of the superficial muscles of the perineum. Global tracking was realized using the modified Gibbs algorithm^7^. The method uses a Gibbs point process framework at its core, using a simulated annealing algorithm that is based on a Monte Carlo dynamics for finite point processes to avoid local minima^29^. The optimization is an iterative process, repeated with an order of 10^8^ iterations. In brief, the reconstructed fibers are built by small line segments (initially hundreds in each voxel), where each segment represents a contribution to the observed diffusion signal. The contribution can be understood as a template signal, originating from a single fiber bundle. In a recent study^7^, the so called “stick model” was used.

The Gibbs method has proven to outperform other existing tracking methods in a Tractography competition based on a realistic phantom made up of the artificial fibers^30^. Moreover, the method is computationally affordable, unlike other global techniques, which may take several days to weeks to complete. The fiber reconstruction itself was applied using the following parameters: 5 × 10^8^ iterations, start and end temperatures of Y = 0.1, T = 0.001. The cylinder parameters were defined as length = 1.172 mm, width = 0.3 mm, 0.096 weight and a 0.2 density penalty parameter (the same parameters were used for the other reconstructions in both males and females, with minor adjustments). All the simulations were run on a Dell Precision T7910, Dual Intel Xeon Processor E5-2687W v4, NVIDIA Quadro M6000 24GB, 256GB RAM. Tractography was carried out using the Fibertools software package (www.uniklinik-freiburg.de/mr/live/arbeitsgruppen/diffusion/fibertools_en.html). Furthermore, to quantitatively describe the fiber orientations in the perineal body and prove their heterogeneity; for each subject, we parametrized the curves passing through the perineal region using a cosine series representation (34).

### Micro CT Imaging

one male (age 57) human cadaver was procured from the University of California, San Diego Anatomical Service. The magnetic resonance imaging (MRI) of the entire pelvis was first performed to identify the region of interest. The anal canal was split in the mid coronal plane into ventral and dorsal halves to further reduce the sample size. The fixed cadaver sample was immersed in 3% PTA until μCT scans confirmed that the contrast had completely penetrated the sample (12 weeks). Samples were finally imaged at (9 or 18 μm)^3^ isotropic voxel size. Precautions were taken to prevent drying of the sample prior to and during the scanning. Imaging was performed on a μCT scanner, Skyscan 1076 (Kontich, Belgium) applying an electrical potential of 100 kVp and current of 100 μA, using a 0.038 mm copper +0.5 mm aluminum filter. From the μCT image data, 2D orthogonal cross-sections were inspected and images were selected using Dataviewer (Skyscan, Belgium). For the 3D rendering, a 3D texture-based volume rendering approach was employed^31^.

## Results

**Figure 1:**
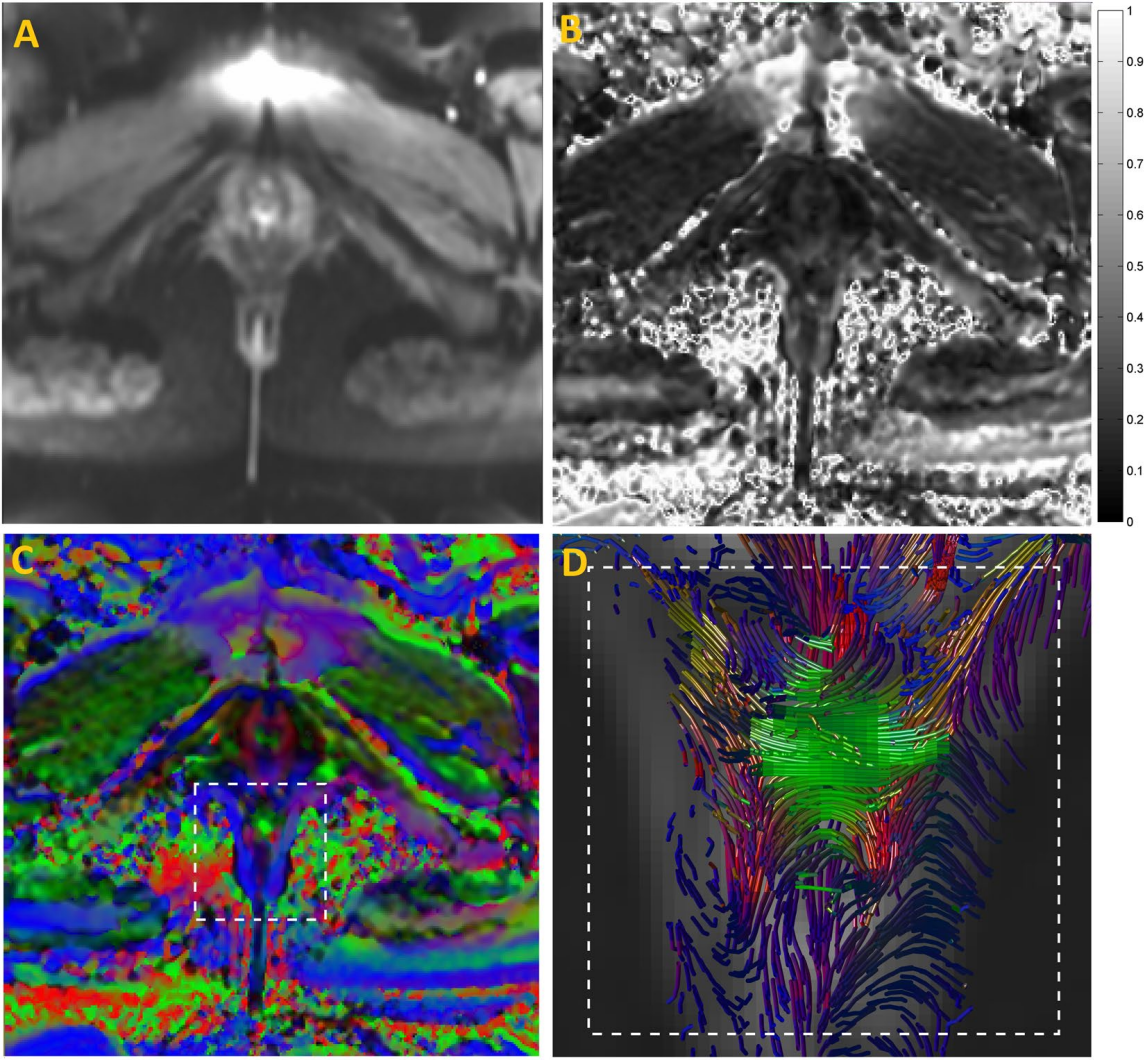
(**A**) Mean DWI image, and (**B**) corresponding FA map of the pelvic floor of a 24-year old, healthy nulliparous female subject (Weight: 58 Kgs., Height: 5′2 ft.), (**C**) direction map with per-voxel color-coded vector values: red (right-left direction), green (antero-posterior direction); blue (cranio-caudal direction). (**D**) Three dimensional (3D) fiber trajectories in the perineal body using FACT algorithm (caudal view).

There were no influencing artefacts and/or distortions detected in the MR diffusion images, thus yielding robust T1W, T2W and DTI data sets. Figure 1 shows the mean DWI images alongside the FA map of a 24 years old nulliparous woman. The per-voxel absolute vector values are color-coded for the direction of muscle fibers: red (anterior-posterior), blue (cranio-caudal) and green (mediolateral). Also shown in Fig. 1D is the resulting tracks in the perineal body of the subject (white dashed rectangle) using the FACT algorithm^32^. Fiber tracking was implemented with the FA threshold set between 0.08 and 0.15, a rather low value, to avoid missing fibers in the low-FA region, and angular threshold between 50° and 70°. Tracks with length under 40 mm were discarded to eliminate fiber fragments. Note, that the bulbospongiosus muscles curves upwards close to the perineal body. The latter is due to the low FA values within the crossing regions; a problem inherent in the local tracking methods that only considers one single fiber at a time thus failing to resolve any possible crossings of fibers within this region.

**Figure 2:**
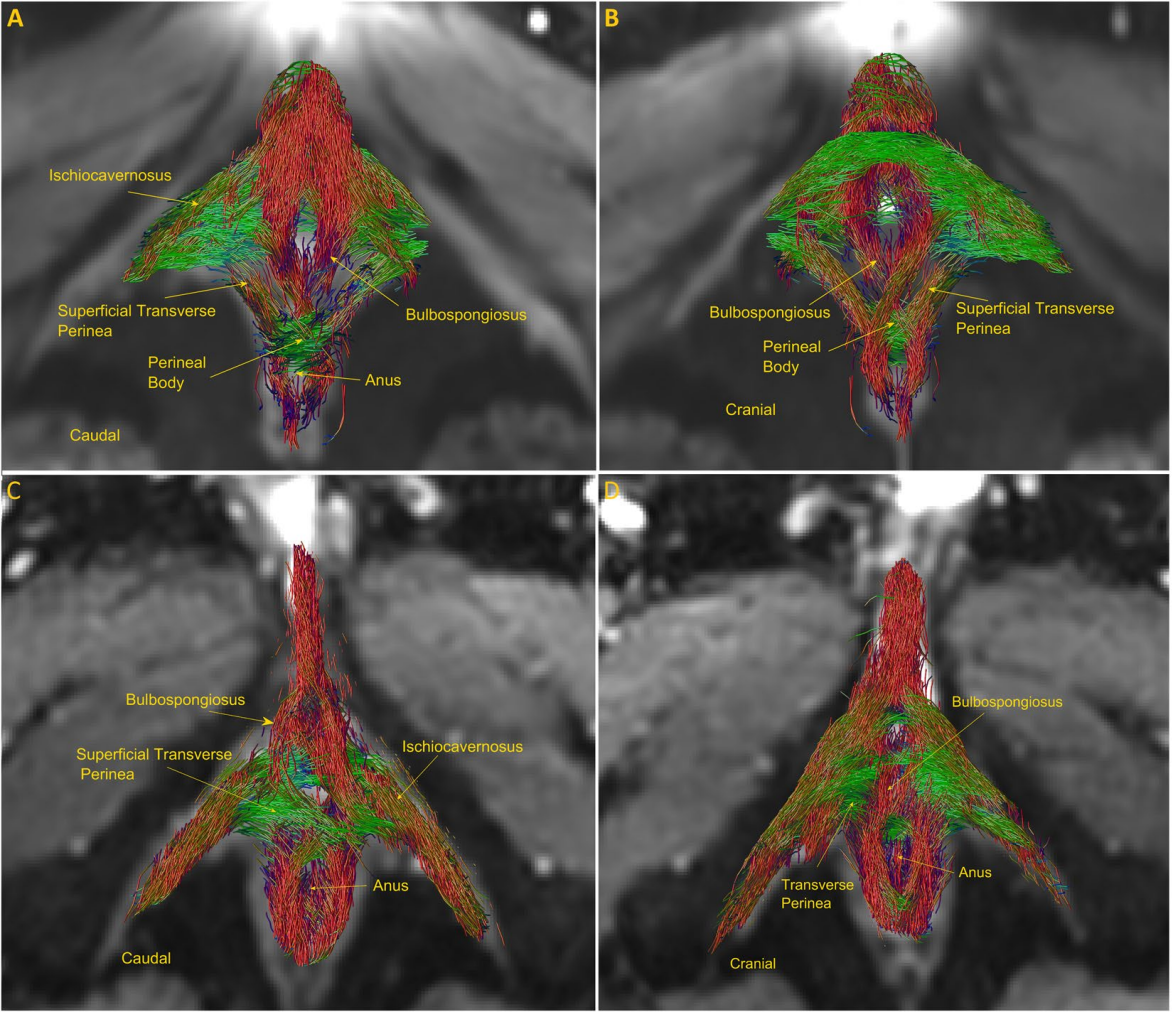
Global reconstruction of the normal subject in Fig. 2 using global fiber tracking (**A**) caudal view, (**B**) cranial view. Note the crossing of muscle fibers in the perineal body, where the transverse perinea and bulbospongiosus muscle fibers meet. (**C**) (superior surface) and (**D**) (inferior surface) show the superficial muscles of perineum in a healthy male subject.

Figure 2A (inferior surface) and 2B (superior surface) shows a sparse global reconstruction of the fibers of superficial muscles of perineum in the same subject as shown in Fig. 1 (See Suppl. Video 1 for a dynamic reconstruction**)**. Note, the crossing of different musculature of the perineum in the perineal body. The figure clearly reveals several muscles: bulbospongiosus, ischiocavernosus, transverse perinea, external anal sphincter and perineal body. The transverse perinea separate the perineum into an anterior (urogenital) and posterior (anal) hiatus. The anterior hiatus, the larger of the two contains the vaginal and urethral orifices. On the other hand, the posterior compartment contains the anal opening. The central point of the perineum or the perineal body lies at the junction of the two compartments. Interestingly, this figure is very similar to the drawing of the superficial muscles of the perineum seen with surgical dissection by several authors^1,3^. Although the origin and insertion point of each muscle is not clearly demonstrated, the generated fibers in each muscle falls within the boundaries of the muscles in the corresponding T2w image series. With regards to the external anal sphincter, the muscle fibers do not loop around the anal canal, they actually decussate in the perineal body, and can be traced into the bulbospongiosus and transversus perinea muscles of the opposite side, which confirms the results reported by several investigators^3,19,33–35^. In other words, the muscles of the external anal sphincter form a “purse-string” like structure.

**Figure 3:**
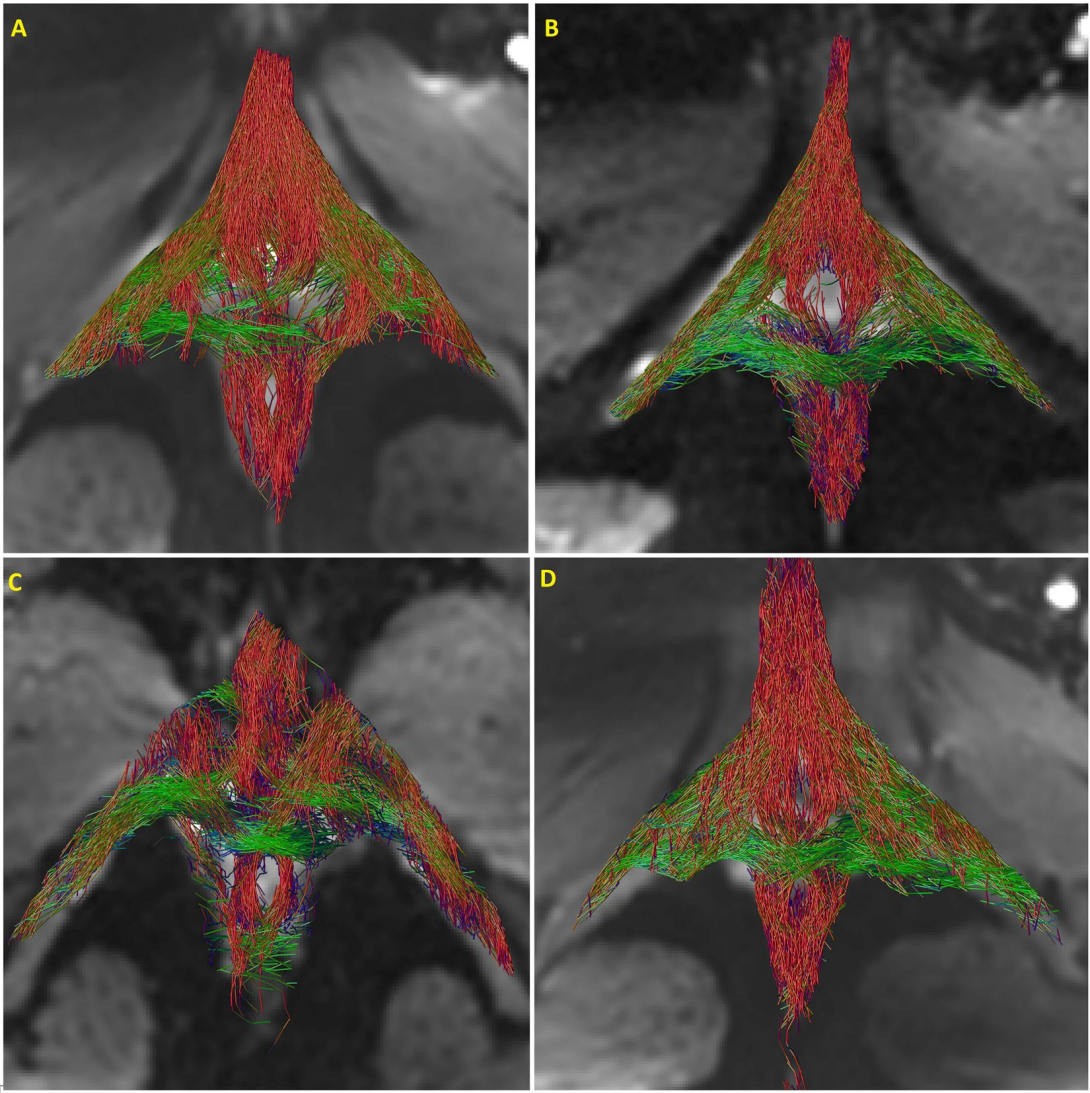
Reconstruction of 4 different female subjects using Global tracking, note, the similarity in the topology across the subjects.

Figure 2C,D show the results of global tractography in a male subject from the superior (cranial) and inferior (caudal) surface. Once again, the anatomy resembles that of the female perineum^1^, and the crossing of muscle fibers at the ventral end of EAS (perineal body). Figure 3 shows the inferior surface of the perineum from 4 females in the dataset. In fact, all 10 female and 4 male subjects reveal consistent morphology confirming the reproducibility of the technique.

**Figure 4:**
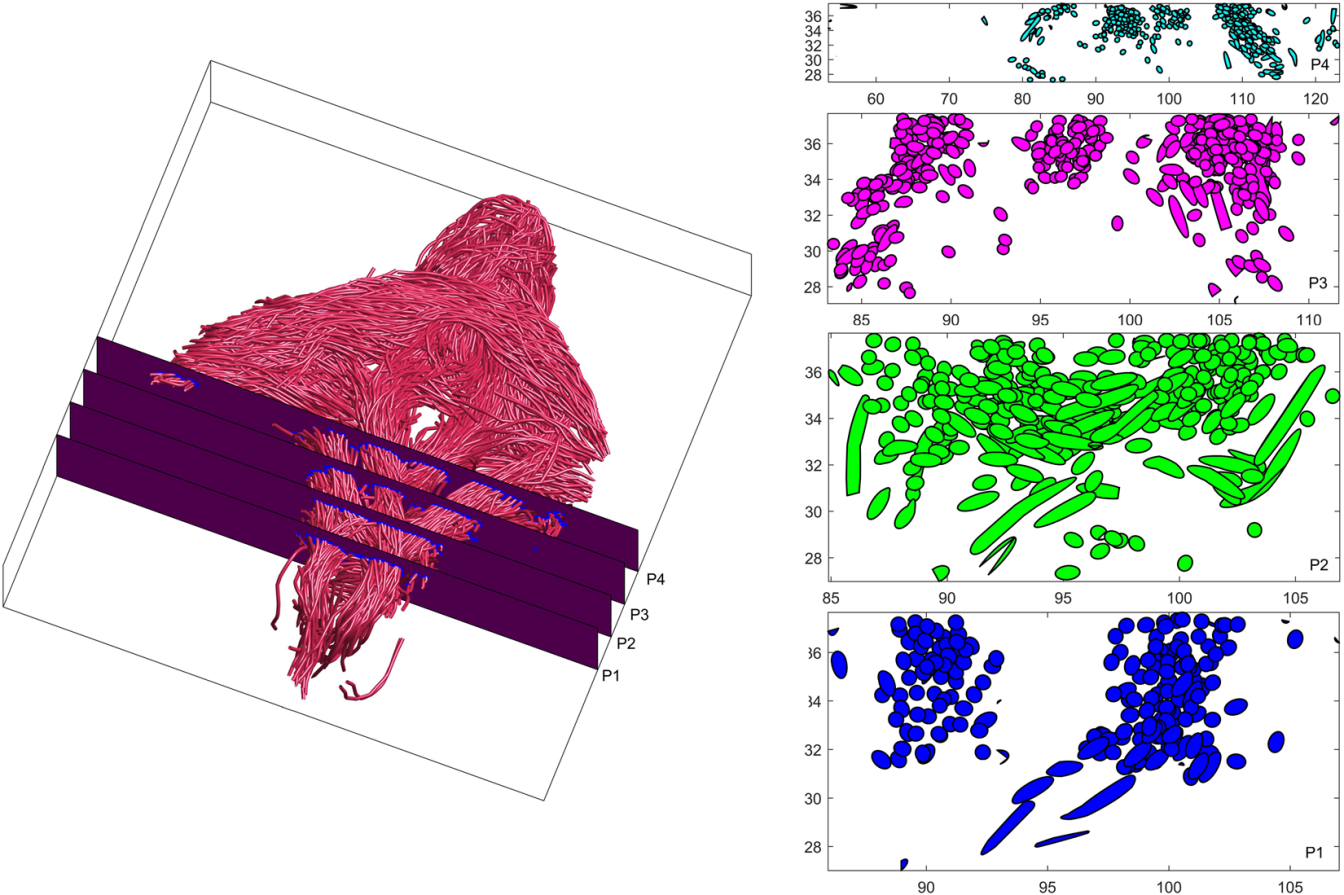
Slicing the fiber trajectories across different regions in proximity of the perineal body. Note, how the fibers get closer moving from the most cranial slice plane (p4). It is also worth noting the direction and area distribution of the p2 slice, where the different muscle groups meet, indicating a heterogeneous distribution of fiber angles within the perineal body.

**Figure 5:**
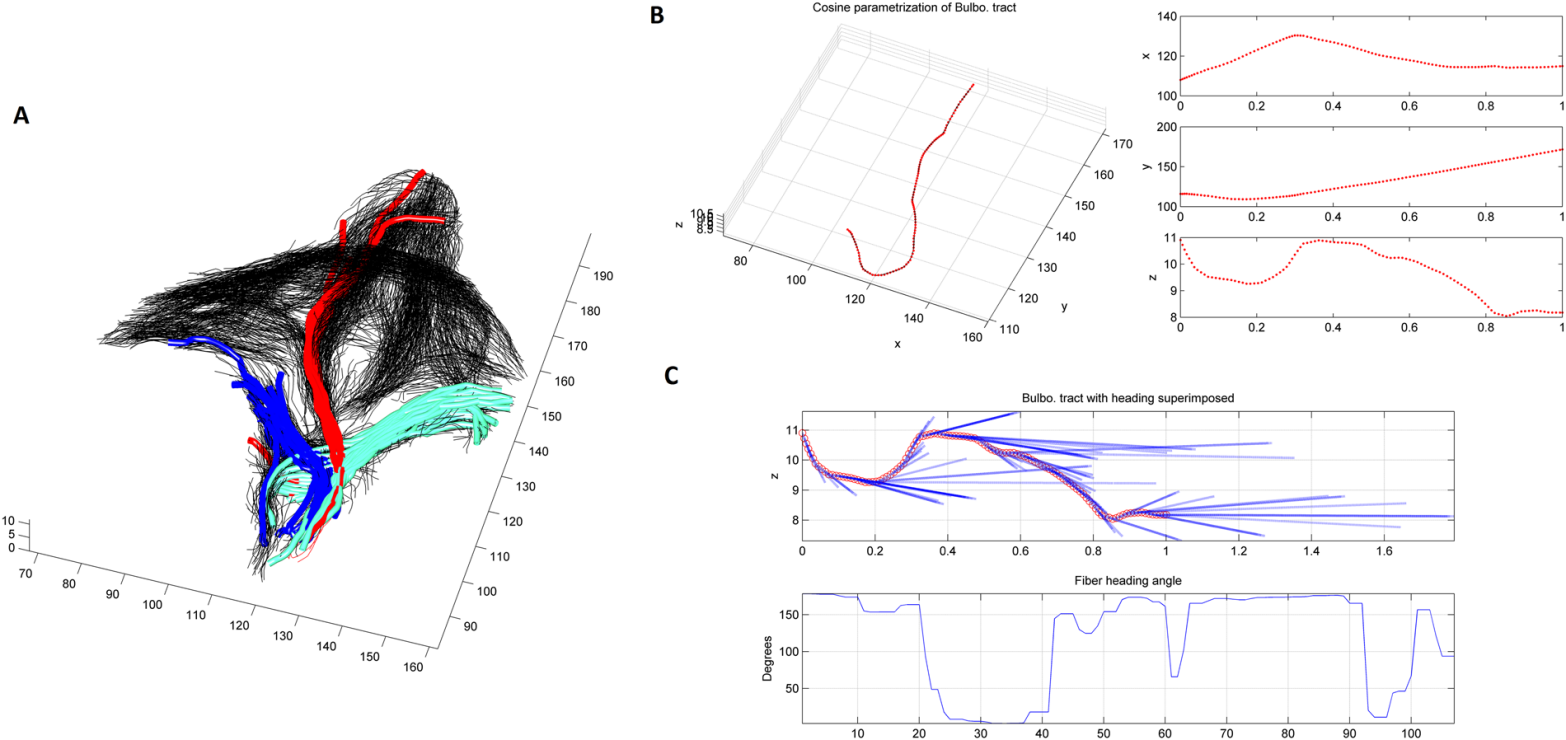
Fiber morphology passing through the perineal body, (**A**) shows several fiber tracts in the bulbospongiosus (red), and the left (cyan) and right (blue) transverse perinea muscles passing through the perineal body, (**B**) Cosine parametrization of a single fiber tract and its subsequent project on the x-,y-, and z- axis, (**C**) calculating the heading direction and curvature of each projection.

**Figure 6:**
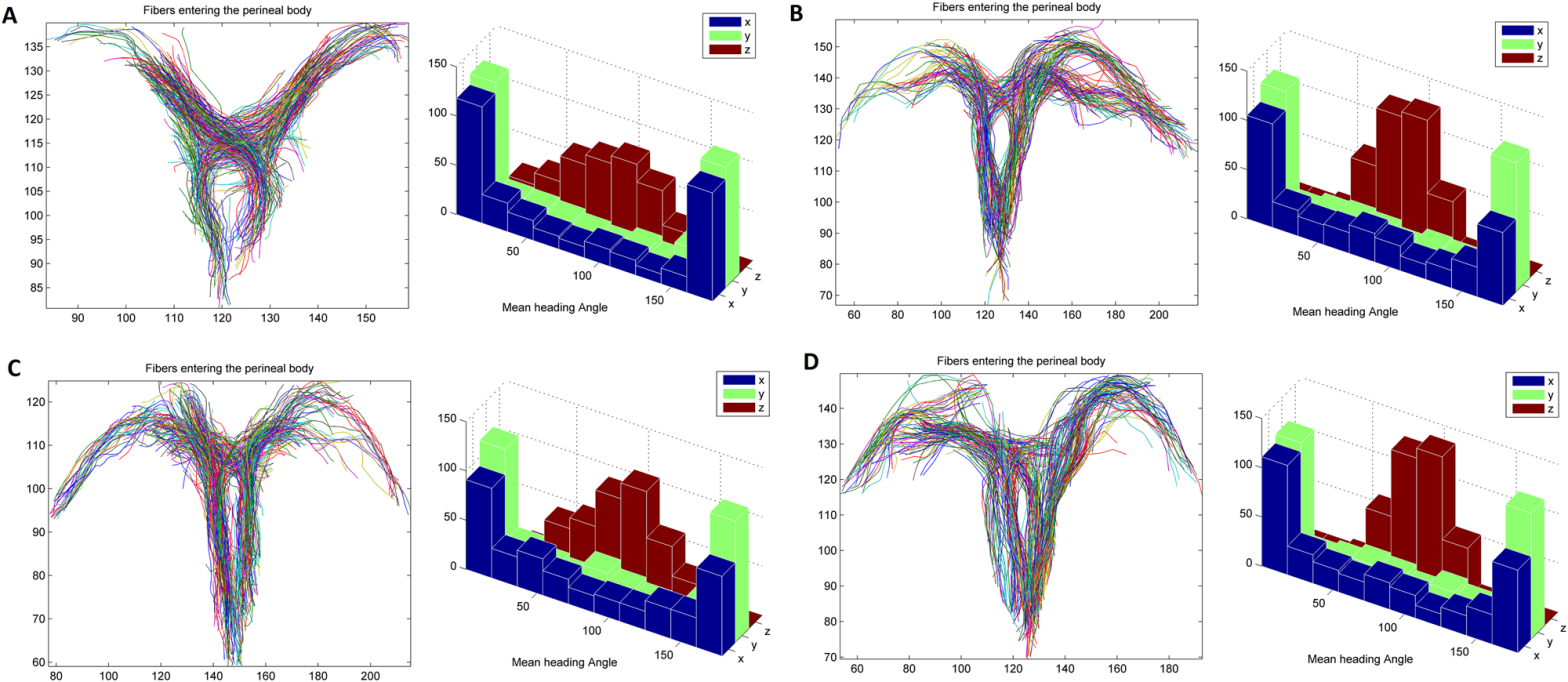
Quantification of the mean fiber heading direction angle of the left and right transverse perinea for 4 healthy females. Each panel shows the clustered tracks, alongside the histogram of mean angular distribution of the mean heading direction in each of the x, y and z-axis.

In order to get a more in depth view of the fiber architecture of the perineum, the reconstructed fibers were sliced across different orthogonal planes. The results of such slicing is shown in Fig. 4. It is worth noting the direction and area distribution of the p2 slice, where different muscle groups meet, indicating a heterogeneous distribution of fiber angles within the perineal body. Next, we parametrize the curves passing through the perineal region using a cosine series representation^36^. The coordinates of curves are parameterized as coefficients of a cosine series expansion, where the spline control points are fitted with the cosine representation with various degrees to the fiber tracts. The latter is shown in Fig. 5B for a sample of the bulbospongiosus muscle. Once each reconstructed 3D tract was projected onto different x, y, z planes, the heading direction, which is the trajectory tangent along each point of fiber curve was calculated, as shown in Fig. 5C. To quantify the heterogeneity of the muscles bundles crossing the perineal body, the left and right transverse perinea muscles were used. The transverse perinea bundles were segmented out from the rest of the fibers using the spectral clustering method (a method for grouping data using eigenvectors of a data affinity matrix) described by O’Donnell^37^. In the latter approach, each tract is embedded as a point in 2D space, where the distance between points is related to their shape similarity. A sample result for 4 of the female subjects is shown in Fig. 6, which was observed in all subjects. Note, the near reflective symmetry of the mean fiber heading direction distribution, around 90 degrees in each of the x, y and z-axes, proving the heterogeneity of incoming fibers into the perineal body. These incoming fibers belong to the bundles of the left and right transverse perinea muscles as they enter the perineal body region.

**Figure 7:**
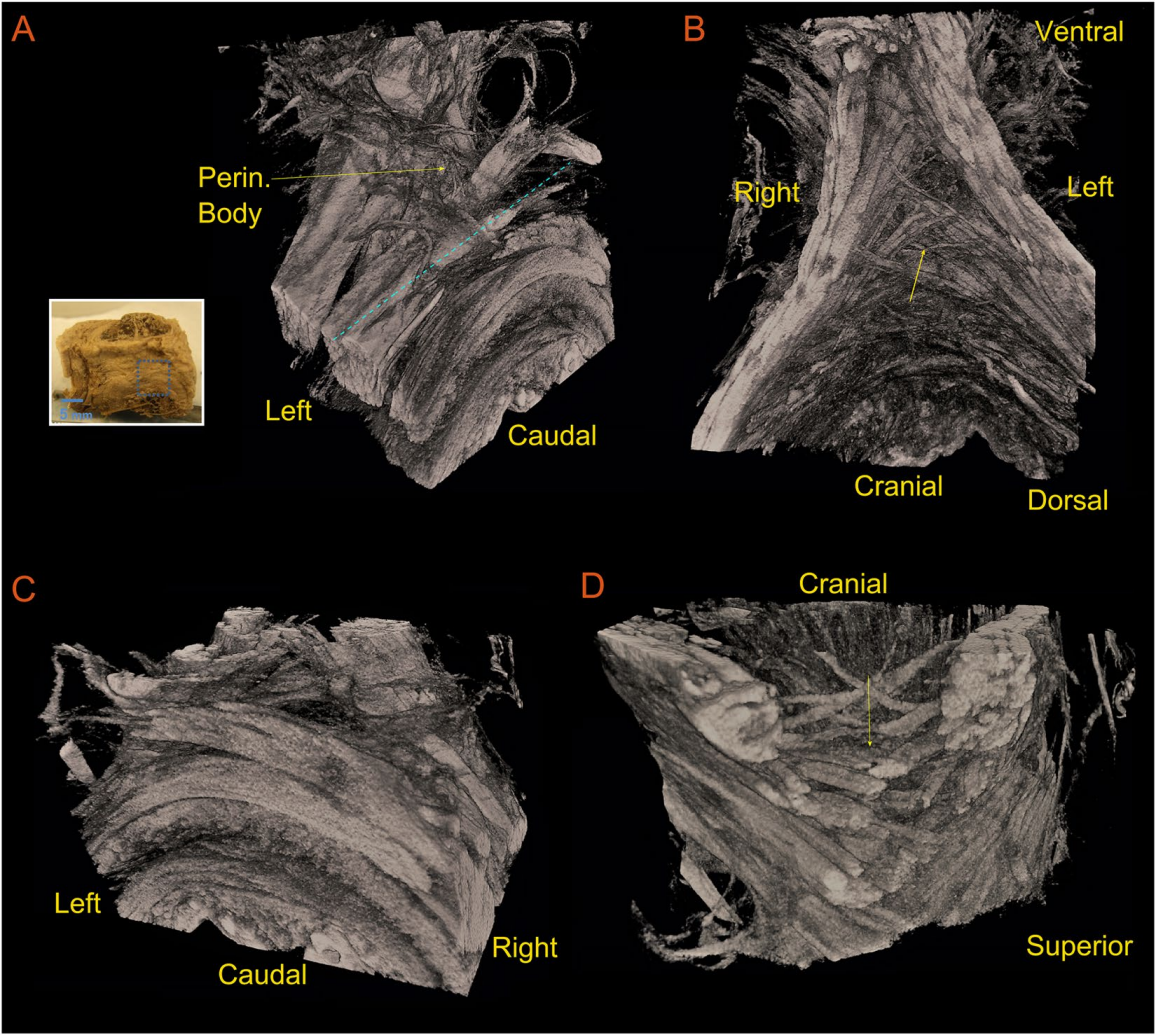
Micro CT of the perineal body in a male cadaver, (**A**) perspective view of the specimen, (**B**) cranial (superior) view showing the area of crossing of fibers right above the anal canal opening, (**C**) the specimen sliced parallel to the xy-plane at the level shown as a dashed blue line in panel (**A**), and finally (**D**) shown a superior view of the sliced specimen, showing the crossing of the fiber bundles, merging toward the ventral midline of the specimen.

Figure 7 shows the region of the perineum containing perineal body from a male cadaver imaged by the micro CT scan. The external anal sphincter muscles from the right and left side of the anal opening cross in the perineal body to the opposite side to continue as bulbospongiosus muscle. Panel B shows a superior (cranial) view of the specimen, where the crossing can be clearly seen. The latter is also the case when the sample is sliced parallel to the x and y plane. The superior view in Panel D (yellow arrow) clearly shows the large region of fiber crossing within the perineal body. This finding is very similar to the crossing pattern seen in the global tractography results of the male subject shown in Fig. 2C,D.

## Discussion

In summary, our results show that using DTI and global fiber tracking methods: (1) all superficial muscles of perineum can be correctly identified in the live humans, in both males and females, and (2) fiber orientation in the individual muscles of perineum can also be correctly determined. We observed crossing of muscles of the EAS from right and left side in the perineal body, and their continuation as transverse perinea and bulbospongiosus muscles. Micro CT images from one male cadaver provided additional support for the conclusion that the muscle fibers in the perineal body cross midline, from one side of the body to the other.

Technical difficulties of imaging might have been one of the reasons why there are a few studies exploring the myoarchitecture of the human pelvic floor using the DTI technique^19,21,22^. Zijta *et al*.^21,22,24^ used DTI with fiber tractography to depict normal pelvic floor anatomy, and to further evaluate pelvic organ prolapse. Although several clinically relevant pelvic structures (including the perineal body, anal sphincter complex, internal obturator muscle and the puboperineal muscle) were identified, no significant differences in the DTI parameters were found between the prolapse group, and the asymptomatic group. The crossing of muscle fibers in the perineal body was not described in any of the above studies. It is very likely that the use of local fiber tracking algorithm in these studies, and the restrictions that come with it, as discussed earlier, failed to resolve crossing/kissing of muscle fibers in the perineal body where several muscle groups meet. Although deterministic fiber tracking approaches are relatively simpler and faster than global methods, estimating the orientation at every voxel in small successive steps may not be entirely accurate. Within a volume, each voxel has a range of potential orientations (such as those encountered in the perineum), and a deterministic tracking method considers the mean of these to be the real orientation. There is a standard error associated with this mean, and these errors are propagated throughout the tract. Therefore, considerable uncertainty in the resulting fiber trajectory can result when using deterministic fiber tracking approach. Local fiber tracking approaches have difficulties with crossing or branching pathways or with pathways in regions of low anisotropic diffusion. In contrast, global approaches consider a larger subspace of the diffusion tensor field at once to infer global characteristics of the diffusion process and to model uncertainty in the diffusion data. They reconstruct the fiber paths simultaneously, while finding the configuration that minimizes the difference between the measured data and the reconstruction. This approach is more stable in the presence of noise and imaging artifacts in the data, a neighborhood of voxels, rather than a single one. However, the above advantage also creates a main disadvantage, which is the higher computational times to produce the tracts. The latter can be reduced though, by knowing the delineating anatomy of the target muscles which requires anatomical knowledge to appropriately define the search space with in the bounding box.

The crossing of muscle fibers in the perineal body has been mentioned by several investigators but its implications and significance in the context of sphincter function has not been fully understood^3^. The sphincters in general are considered to be “donut” or “ring” shaped muscle and accordingly the EAS is thought of as a circular muscle. Current thinking is that the muscles from the right and left sides of the EAS are attached to the fibro-tendinous midline structure, i.e., perineal body. The latter is also considered to be the insertion site for transverse perinea and bulbospongiosus muscles of the perineum. For the first time, using micro CT imaging technique, we show the crossing of muscle fibers in the perineal body of a male cadaver. The results of our DTI based fiber tractography confirms the crossing of muscle fibers in the perineal body.

The superficial and deep muscles of the pelvic floor are frequently damaged during vaginal child birth related injuries resulting in anal incontinence, urethral incontinence and pelvic organ prolapse. Endoanal ultrasound imaging is the current gold standard to diagnose injury to the anal sphincter muscle, which assumes circular morphology of the external anal sphincter. Bulbospongiosus, and transverse perinea muscles are not generally assessed with US imaging and therefore it is difficult to fully image the “purse string” morphology of the EAS using US approach. Current corrective surgical technique, i.e., sphincteroplasty, restores the circular shapes of EAS. If the EAS is in fact shaped like a “purse string”, both diagnostic and corrective techniques to restore the EAS function are most likely inaccurate, which may be the reason for poor outcome of the corrective surgery^38^. Furthermore, based on the “purse string” morphology of the EAS^19^, one can understand why neither a lateral episiotomy, which sections through transverse perinea and bulbospongiosus muscle, nor a midline episiotomy that sections through the perineal body are sphincter sparing operations.

The limitations of our study are, (1) the number of subjects studied were relatively small, 10 females and 4 males. However, we believe that the goal of our study was limited, and we were successful in achieving those goals. (2) We only studied one male cadaver using micro-CT imaging technique. The reason for the above was that childbirth related injuries are extremely common in women, and we could not procure a nulliparous female cadaver. Micro-CT imaging technique is quite cumbersome; it takes long time to achieve the final result. We did not want to risk conducting micro-CT imaging and fail to make a definitive conclusion about crossing of the muscle fibers in the perineal body.

In conclusion, we propose that the DTI-based global fiber tractography is a promising technique to image the muscles of EAS complex, including perineal body to accurately identify the injury to the muscles of EAS complex and superficial muscles of perineum.

## Electronic supplementary material

Supplementary Video
